# Another potential carp killer?: Carp Edema Virus disease in Germany

**DOI:** 10.1186/s12917-015-0424-7

**Published:** 2015-05-15

**Authors:** Verena Jung-Schroers, Mikolaj Adamek, Felix Teitge, John Hellmann, Sven Michael Bergmann, Heike Schütze, Dirk Willem Kleingeld, Keith Way, David Stone, Martin Runge, Barbara Keller, Shohreh Hesami, Thomas Waltzek, Dieter Steinhagen

**Affiliations:** Fish Disease Research Unit, University of Veterinary Medicine, Hannover, Germany; Friedrich-Loeffler-Institut, Federal Research Institute for Animal Health, Institute of Infectology, Greifswald, Germany; Lower Saxony State Office for Consumer Protection and Food Safety, Veterinary Task-Force, Hannover, Germany; Centre for Environment, Fisheries, and Aquaculture Science (CEFAS), Weymouth, Dorset, UK; Lower Saxony State Office for Consumer Protection and Food Safety, Food and Veterinary Institute Braunschweig/Hannover, Hannover, Germany; Department of Infectious Diseases and Pathology, University of Florida, College of Veterinary Medicine, Gainesville, FL USA

**Keywords:** Carp Edema Virus, Koi sleepy disease, *Cyprinus carpio*

## Abstract

**Background:**

Infections with carp edema virus, a pox virus, are known from Japanese koi populations since 1974. A characteristic clinical sign associated with this infection is lethargy and therefore the disease is called “koi sleepy disease”. Diseased koi also show swollen gills, enophthalmus, and skin lesions. Mortality rates up to 80 % are described. For a long period of time, disease outbreaks seemed to be restricted to Japan. However, during the last years clinical outbreaks of koi sleepy disease also occurred in the UK and in the Netherlands.

**Case presentation:**

In spring 2014 koi from different ponds showing lethargic behavior, skin ulcers, inflammation of the anus, enophthalmus, and gill necrosis were presented to the laboratory for diagnosis. In all cases, new koi had been purchased earlier that spring from the same retailer and introduced into existing populations. Eleven koi from six ponds were examined for ectoparasites and for bacterial and viral infections (cyprinid herpesviruses in general and especially koi herpesvirus (KHV) known formally as *Cyprinid herpesvirus 3* (CyHV–3); and Carp Edema Virus). In most of the cases parasites were not detected from skin and gills. Only opportunistic freshwater bacteria were isolated from skin ulcers. In cell cultures no cytopathic effect was observed, and none of the samples gave positive results in PCR tests for cyprinid herpesviruses. By analyzing gill tissues for CEV in seven out of eleven samples by a nested PCR, PCR products of 547 bp and 180 bp (by using nested primers) could be amplified. An outbreak of Koi Sleepy Disease was confirmed by sequencing of the PCR products. These results confirm the presence of CEV in German koi populations.

**Conclusion:**

A clinical outbreak of “koi sleepy disease” due to an infection with Carp Edema Virus was confirmed for the first time in Germany. To avoid transmission of CEV to common carp testing of CEV should become part of fish disease surveillance programs.

**Electronic supplementary material:**

The online version of this article (doi:10.1186/s12917-015-0424-7) contains supplementary material, which is available to authorized users.

## Background

Virus infections are responsible for serious diseases associated with high morbidity and mortality in fish [1–3]. In common carp and ornamental koi (*Cyprinus carpio*), *Cyprinid herpesvirus 3* (CyHV–3, KHV), is considered as major viral pathogen. It causes a notifiable disease (KHVD) for OIE [4] and EU [5] which is associated with skin lesions and gill necrosis [2]. Since its first appearance in the late 1990s it seriously affects carp aquaculture, wild carp, and koi trade worldwide. In addition, infections with carp edema virus (CEV), a poxvirus, are known to cause health problems in koi apparently with geographically restricted incidence [6, 7]. CEV infections have been observed in Japan since 1974, mainly in spring and autumn at water temperatures between 15 °C and 25 °C, when koi are moved from earthen ponds to freshwater containing cement-lined ponds [6]. In adult fish, lethargic behavior is a characteristic clinical sign associated with this infection and therefore the disease was named “koi sleepy disease” (KSD). Other typical pathological symptoms include swollen gills [6], enophthalmus and skin lesions, often around the mouth, and at the base of the fins [7]. Mortality rates up to 80 % were observed, without the presence of parasites, fungi, or high levels of fish pathogenic bacteria in diseased koi [3]. For decades KSD outbreaks seemed to be restricted to Japan, but during the last years CEV infections associated with clinical outbreaks of KSD occurred in defined koi populations in the UK [7] and in the Netherlands [8]. In the present study, a clinical outbreak of KSD due to an infection with CEV was confirmed in spring 2014 for the first time in koi in Germany. The outbreak originated from one source, involved several koi populations in different ponds and confirmed the pathogenic nature of the virus.

## Case presentation

**Fig. 1:**
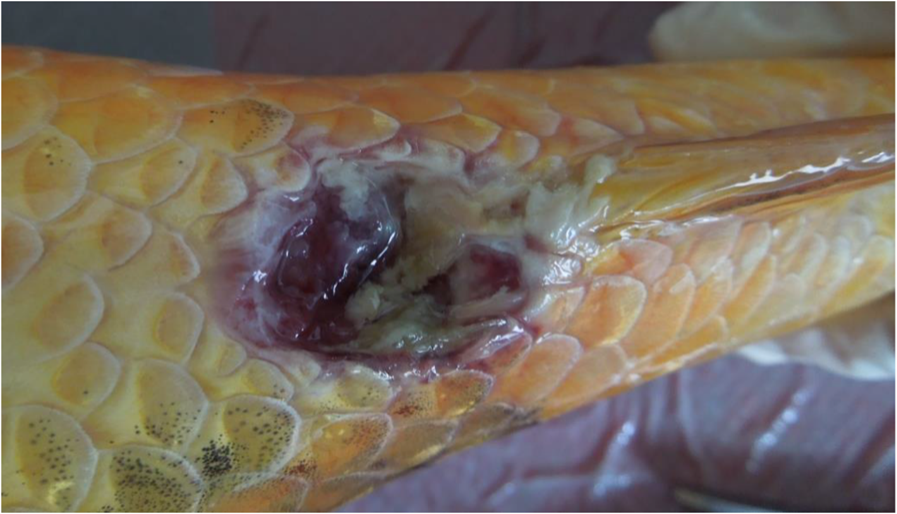
Koi of this study showing an ulcerative inflammation of the anus. The koi was CEV-infected

**Fig. 2:**
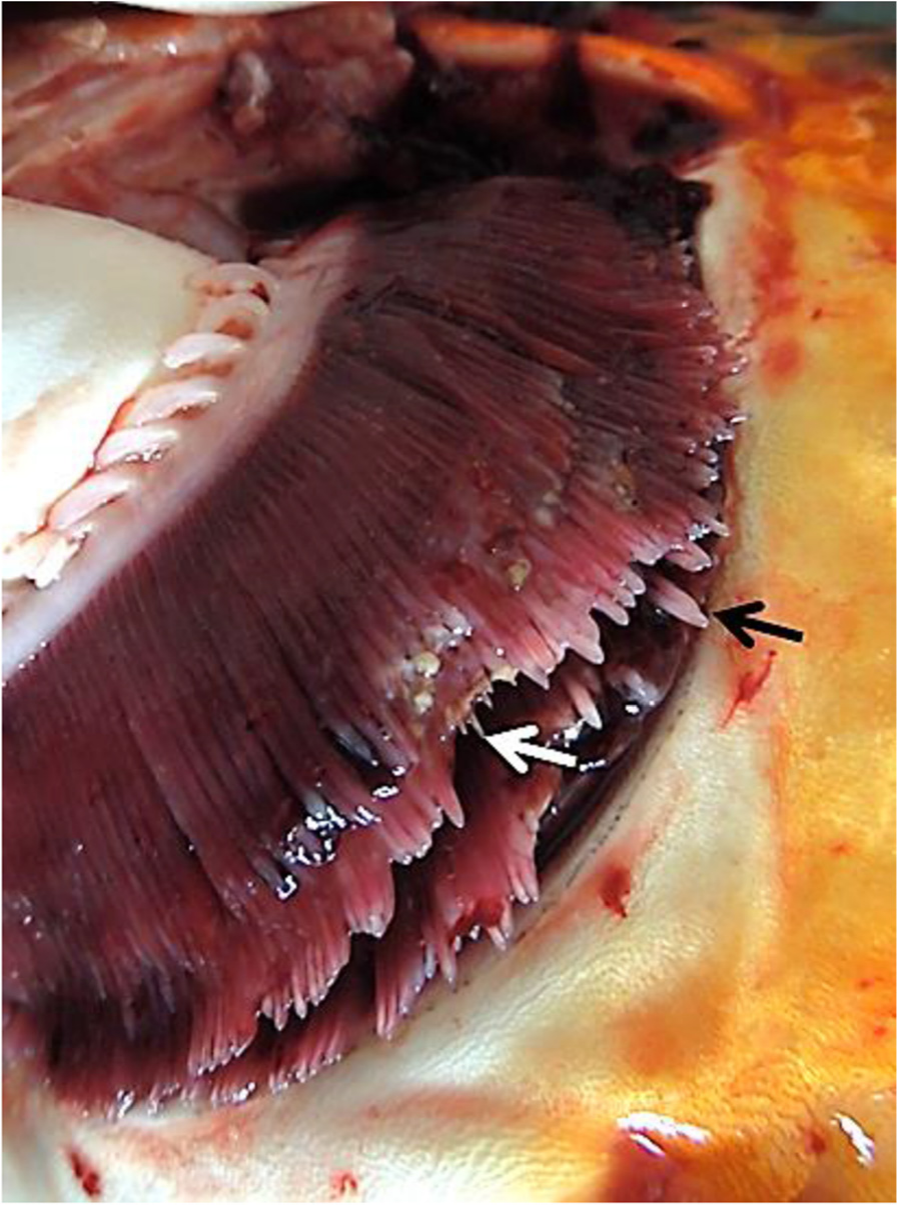
Gills of CEV-infected Koi. Swelling of the primary filaments (black arrow) and necrosis of gill tissue (white arrow) can be seen

In spring 2014 several koi hobbyists presented koi showing similar clinical signs to the consulting service of the Fish Disease Research Unit at the University of Veterinary Medicine in Hannover, Germany. In most cases the koi displayed the following clinical signs: lethargy, ulcerations on the mouth, or on the lateral side of the body, a reddened inflamed anus (Fig. 1), enophthalmus and gill swelling, or gill necrosis (Fig. 2) affecting up to one third of the gill tissue. In one pond all koi died within three days without showing disease symptoms. In all ponds the water temperature ranged between 17 °C and 22 °C. A failure of the filter systems was excluded as cause of death, as water analyses showed no alterations. In all cases new koi had been purchased in spring 2014 from the same retailer. In affected ponds newly purchased koi as well as koi kept already for a couple of years developed clinical symptoms. Other fish species, like goldfish, and sturgeons, kept in the same ponds were not affected. Hence an infectious agent was suspected to be responsible for the disease outbreaks.

In total 11 fish from 6 ponds were examined. Fresh smears of gills and skin of all koi were inspected microscopically for ectoparasites. Bacteriological examinations were performed by routine cultivation methods by taking swabs from skin ulcerations and from internal organs. Gill tissues from humanely euthanized koi were fixed in 4 % buffered formalin, embedded into paraffin, cut, and stained with hematoxylin and eosin (HE staining) for histological evaluation [9]. For virological examination, samples of gills, kidney, gut, and brain were collected. Samples from 3 fish from different ponds were inoculated on Fathead Minnow (FHM) cells at 22 °C and on Common Carp Brain (CCB) cells at 25 °C for two passages of each 7 to 10 days according to standard procedures [10]. From all fish, DNA was isolated from tissue pools as well as from separate gill samples by DNA isolation kits (Qiagen, Germany, Macherey & Nagel, Germany). Samples were analysed for the presence of DNA sequences specific for cyprinid herpesviruses in general by an end point PCR described by Engelsma et al. [11] using KAPA2G Robust Hot Start PCR kit (Peqlab, Germany). For CyHV–3 samples were tested by real time PCR according to Gilad et al. [12] using Quantitect Multiplex qPCR Mix (Qiagen, Germany). DNA from gills was examined for the presence of CEV specific DNA sequences by end point PCR described by Oyamatsu et al. [13].

**Fig. 3:**
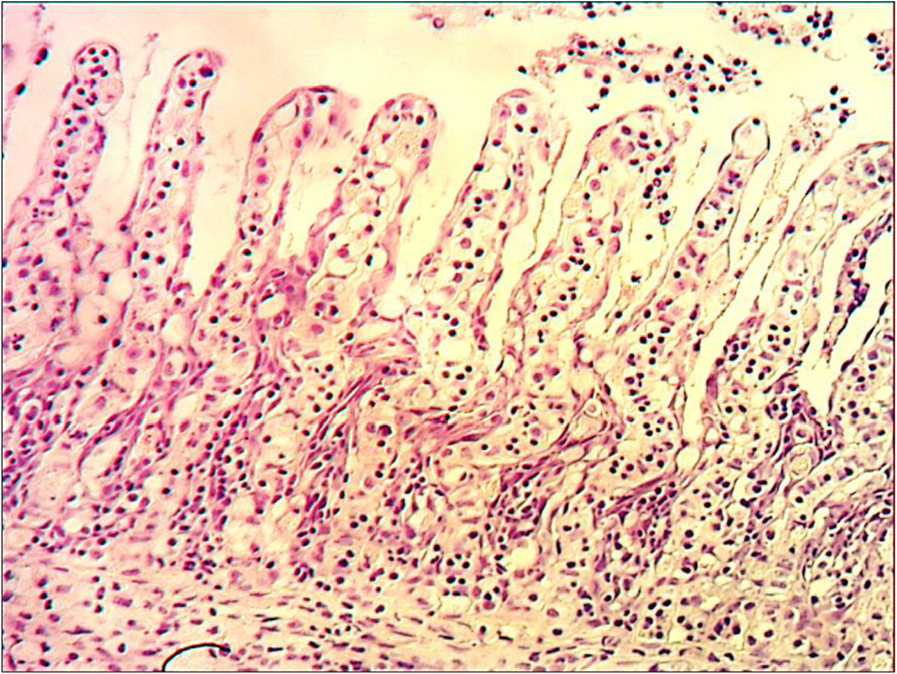
Histopathology of gill tissue of a CEV-infected koi showing cell edema and detachment of epithelial cells. HE staining at 400 × magnification

In 10 cases parasites could not be detected in skin and gill smears. A moderate to high amount of opportunistic freshwater bacteria, in particular *Aeromonas* spp., could be isolated from skin, while in samples taken from internal organs no bacterial growth was found. In the histological examination gill tissue of diseased fish showed hyperplasia and clubbing of primary gill filaments, thickening, and edema of epithelial cells in secondary filaments and a detachment of epithelial cells (Fig. 3).

**Fig. 4:**
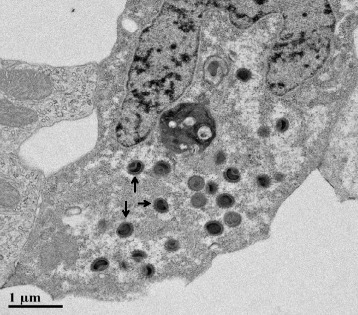
Transmission electron photomicrograph of a koi gill epithelial cell containing large intracytoplasmic spheroid CEV virions indicated by the arrows

Cytopathic effect was not observed in any of the virus isolations. In PCR analyses none of the samples gave positive results for cyprinid herpesviruses, including CyHV–3. By analysing gill tissues for CEV, 7 out of 11 samples showed PCR products of 547 bp and 180 bp (by using nested primers) which were sequenced (LGC Genomics, Berlin, Germany). In the German reference laboratory for koi herpesvirus disease at the Friedrich Loeffler-Institut the sequences were compared to sequences obtained from positive CEV material from the OIE reference laboratory in Weymouth (CEFAS, UK), the Wildlife, and Aquatic Veterinary Disease Laboratory in Gainesville, Florida (WAVDL, USA), and to the sequence published in the PhD Thesis of Oyamatsu [14]. Nucleotide comparisons with the original sequence from Oyamatsu [14] revealed greater than 96 % sequence identity, confirming CEV was detected in this study (Additional file 1). The received sequence was added to gene bank (Gene Bank ID: KM283182). Furthermore, transmission electron microscopy studies performed at WAVDL have revealed that samples from koi positive for CEV by PCR also yield poxvirus-like virions (Fig. 4) within infected gill epithelial cells as has been repeatedly reported [3, 6, 15].

The disease induced by CEV occurs at a temperature range similar to KHVD and also develops similar clinical signs [16]. In particular gill and skin alterations, delineated as characteristic in outbreaks of KHVD [2], were also recorded from clinical KSD [6, 15]. In the cases described here, most affected koi showed profound gill necrosis. In the few PCR positive fish that displayed normal gills during gross examinations, histological changes were noted including hyperplasia of gill tissue consistent with previous reports on CEV [6, 7]. Additionally an inflammation of the anus was detected in many cases. The similarity of the clinical signs of KHVD and KSD complicate diagnosis. During the past years in our practice several cases of mortality in koi associated with gill necrosis were observed without detection of CyHV–3 or a severe gill infection with parasites or bacteria. Some of those cases could be related to an infection with CEV, but this would need testing to confirm.

An infection with a CEV-like virus was also detected in stocks of clinically healthy koi imported from Israel and Japan to the UK [7]. Hence, trading clinically healthy but CEV infected koi might promote spread of KSD, not only to ornamental koi but also to European carp aquaculture. A CEV-like virus was already detected in cultured common carp undergoing mortality and displaying clinical disease in the UK in March 2012 [7].

## Conclusion

This is the first report of CEV detection related to KSD outbreaks in Germany. It further confirms the presence of Carp Edema Virus in European koi populations. We recommend to include the CEV PCR into routine diagnostics.

Fish health services of continental Europe should be aware of the presence of CEV in Europe which may result in high losses in carp aquaculture. Action should be taken to prevent transmission of CEV from koi to common carp. Infections with CEV might be treated as an emerging disease in Germany. Therefore it is considered, if, and what kind of action according to EU directive 2006/88/EG is necessary in the case of koi sleepy disease outbreaks. European countries should avoid the spread of CEV infections in Europe like it happened with KHVD, where surveillance programs were established too late to prevent spread of the disease. Therefore, testing koi, and carp for CEV should become part of fish disease surveillance programs of national and regional fish disease laboratories.

## Additional file

Additional file 1:
**Comparison of the sequence obtained in this study to the sequence published in the PhD Thesis of T. Oyamatsu (Reference 14).**
