# Supplementary material for: Another potential carp killer?: Carp Edema Virus disease in Germany
Source: BMC Vet Res. 2015 May 15;11:114. doi: 10.1186/s12917-015-0424-7 (PMC4431602; doi:10.1186/s12917-015-0424-7)
Supplement: Additional file 1: — Comparison of the sequence obtained in this study to the sequence published in the PhD Thesis of T. Oyamatsu (Reference 14). [file 12917_2015_424_MOESM1_ESM.docx]

1. Sequence from Oyamatsu PhD

>Oyamatsu

TGCAGGATCATTATCCTTGTATCTACAAGTACAATAGAAAGCAAGAAGTTTGTATGGAGT

ATCCAAAGTACTTAGATTAATGTTATCAATGAAATTTGTGTATTGTGTTTTTGTTAGTCC

AAGAGTTTTCTTCTCATCGTTTGTTACCTTTTGTAGTTGTTTAATATTTGTGATAAGATT

TCCATTAGCATAAAATCCTTCCCAAATTTGTGTTGATACATGTTTTAGTGTTTTGTAGAT

TGTAGCATTTCCTAGTTTGTATGGCAAGAAACAAACTCTCTTTACTGCAACTCCTTGAGG

AATTTGATCTAGAATTCCACAGAATGTAATCTCAAATTTGTTTGTAGAGTTTTTGAAGTA

TACTGTTTCATCACACAATCCTAGAACTAGAGCAAGATTAGAAGTCATTGTCTTATCGAA

GACATTCATCTTATTCCAATCATCAATCTGAATTCCTTTCCAGAACATAACATTTGCAAT

TTTAACTTGCTCTGGAATTGTATCAACATGTCCAATATCTTTCTTTACTACGTAATTTGG

ATGAGGTAGTACTTTGCTAACAAAGTCACAATAGTGAAGAGTTGTCATTTTAATTTGTTG

TAGTCCAATTTCTGCAAATTGATATATATCAGGAATATCAAATTTAACCATATTTGCAAA

TGGATTTGCTGCTGGTGCTGCCATTACGTAATTAGAATCGCGAAGTTCAGGATCTCTTGC

TGCTGCTGTTGCAACCATTTGAGAATGAACCGAATCAACAAGTTGATATGCTTTTGCATT

TGCATCAAAAGCAACAACTTGACGAGGGAATGATTGGGACAAAGTAGAACTTCTTGTATA

ATGTATATCTTGAGAAGCAGCTGCTCCACCTGCTACAATTCCAAGAGCATAATGATATTC

AAGATCTAGTTTAATTTGATCTGGAGAATAAGTGTATGCCTTAATTCCATATAGCTTAAT

GAAATGCTCATAATTACCTTGTCCAAACAAACTTAGGTAAAATAGTTTTAGGATTGAAGC

AAGAGCTGCTGCACTTTTAGGAGGACAAGTAAAGTTACCACCAGCTCCCTACAAGGAAAG

CAATTGATTTTATACTTGAAGAACAATCTAGAAGATTGGAGAAATTCTCAAGAATTAGAA

TTGCAACTTCTAGTCTCTCTAGTTTTTCTAGATTTAGATTTAGGTTGGCATCGAAATAAC

TTGCATAATCTAGAAGTTCATCAACATCAAATGTACTTACATCAAATAGGAAAGGATTAG

GAGCAACCTGCA

1. Jung-Schroers et al amplicon

>Amplicon

ATGAACTGAATCAACAAGTTGATATGCATTTGCGTTTGCATCAAAaGTAATAAGTTGACGAGGGAATGATTGAGATAAAGTAGAACTTCTTGTATAATGTATATCTTGAGAAGCAGCTGCTCCACCTGCTACAATTCCAAGAGCATAATGATATTCAAGATCTAGTCTAATTTGATCTGGAGAATAAGTATATGCTTTGATTCCATACAGTTTAATGAAATGCTCATAATTACCTTGTCCAAACAAAGTTAGATAAAATAGTTTTAGGATTGAAGCAAGAGCTGCTGCACTTTTAGGAGGACAAGTAAAGTTACCACCAGCTCCTACAAGGAAAGCAATTGATTTTATACTTGAAGAACATTCAAGAAGATTAGAGAAATTTTCAAGAATTAGAATTGCAACTTCTAGTCTCTCTAGTTTTTCTAGATTTAGATTTAGGTTGGCATCGAAATAACTTGCATAATCTAGAAGTTCATCAACATCAAATGTACTTACATCAAATAGGAA

1. Translation of sequence from the PhD Thesis of Takeshi Oyamatsu

[3'5' Frame 1](http://web.expasy.org/cgi-bin/translate/dna_sequences?/work/expasy/tmp/http/seqdna.9408,4)
CRLLLILSYL**MStop**VHL**M**L**M**NF**Stop**I**M**QVIS**M**PT**Stop**I**Stop**I**Stop**KN**Stop**RD**Stop**KLQF**Stop**FLRISPIF**Stop**IVLQV**Stop**NQLLSL**Stop**GAGGNFTCPPKSAAALASILKLFYLSLFGQGNYEHFIKLYGIKAYTYSPDQIKLDLEYHYALGIVAGGAAASQDIHYTRSSTLSQSFPRQVVAFDANAKAYQLVDSVHSQ**M**VATAAARDPELRDSNYV**M**AAPAANPFAN**M**VKFDIPDIYQFAEIGLQQIK**M**TTLHYCDFVSKVLPHPNYVVKKDIGHVDTIPEQVKIANV**M**FWKGIQIDDWNK**M**NVFDKT**M**TSNLALVLGLCDETVYFKNSTNKFEITFCGILDQIPQGVAVKRVCFLPYKLGNATIYKTLKHVSTQIWEGFYANGNLITNIKQLQKVTNDEKKTLGLTKTQYTNFIDNINLSTLDTPYKLLAFYCTCRYKDNDPA

1. Protein BLAST results confirming that our sequence is similar to other poxvirus P4a core protins


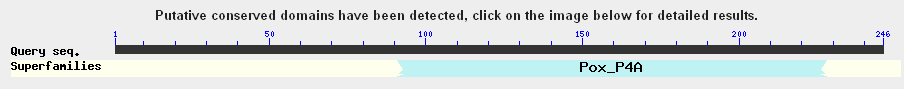


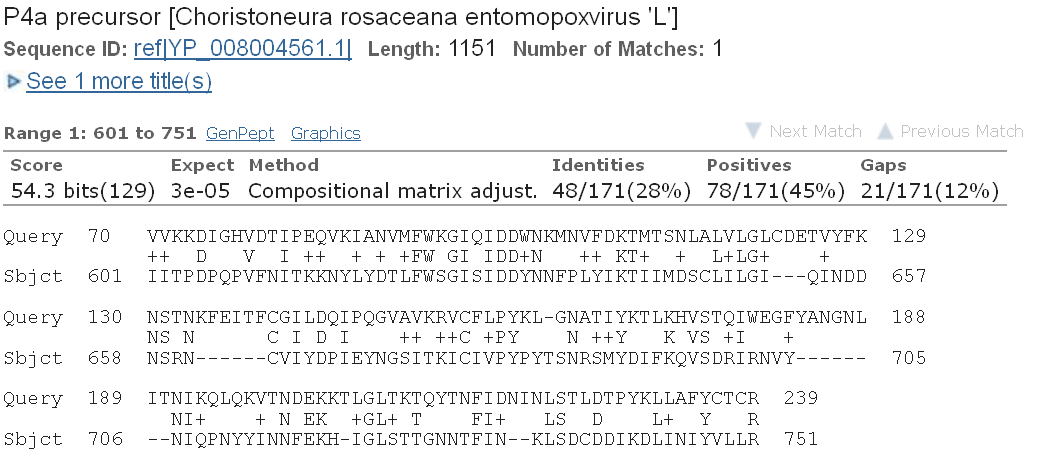


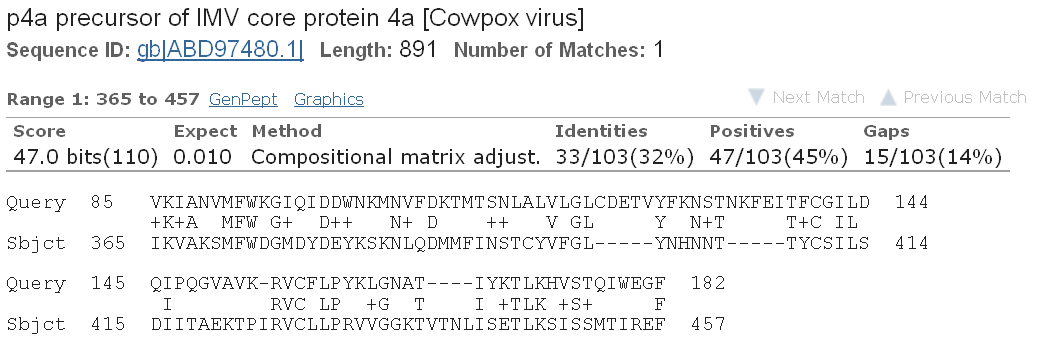


1. CLUSTALO(1.2.1)multiple sequence alignment - Identity 96% (Oyamtatsu 1996 vs. Our sequence)

OyamatsuTGCAGGATCATTATCCTTGTATCTACAAGTACAATAGAAAGCAAGAAGTTTGTATGGAGT

Amplicon------------------------------------------------------------

OyamatsuATCCAAAGTACTTAGATTAATGTTATCAATGAAATTTGTGTATTGTGTTTTTGTTAGTCC

Amplicon------------------------------------------------------------

OyamatsuAAGAGTTTTCTTCTCATCGTTTGTTACCTTTTGTAGTTGTTTAATATTTGTGATAAGATT

Amplicon------------------------------------------------------------

OyamatsuTCCATTAGCATAAAATCCTTCCCAAATTTGTGTTGATACATGTTTTAGTGTTTTGTAGAT

Amplicon------------------------------------------------------------

OyamatsuTGTAGCATTTCCTAGTTTGTATGGCAAGAAACAAACTCTCTTTACTGCAACTCCTTGAGG

Amplicon------------------------------------------------------------

OyamatsuAATTTGATCTAGAATTCCACAGAATGTAATCTCAAATTTGTTTGTAGAGTTTTTGAAGTA

Amplicon------------------------------------------------------------

OyamatsuTACTGTTTCATCACACAATCCTAGAACTAGAGCAAGATTAGAAGTCATTGTCTTATCGAA

Amplicon------------------------------------------------------------

OyamatsuGACATTCATCTTATTCCAATCATCAATCTGAATTCCTTTCCAGAACATAACATTTGCAAT

Amplicon------------------------------------------------------------

OyamatsuTTTAACTTGCTCTGGAATTGTATCAACATGTCCAATATCTTTCTTTACTACGTAATTTGG

Amplicon------------------------------------------------------------

OyamatsuATGAGGTAGTACTTTGCTAACAAAGTCACAATAGTGAAGAGTTGTCATTTTAATTTGTTG

Amplicon------------------------------------------------------------

OyamatsuTAGTCCAATTTCTGCAAATTGATATATATCAGGAATATCAAATTTAACCATATTTGCAAA

Amplicon------------------------------------------------------------

OyamatsuTGGATTTGCTGCTGGTGCTGCCATTACGTAATTAGAATCGCGAAGTTCAGGATCTCTTGC

Amplicon------------------------------------------------------------

OyamatsuTGCTGCTGTTGCAACCATTTGAGAATGAACCGAATCAACAAGTTGATATGCTTTTGCATT

Amplicon------------------------ATGAACTGAATCAACAAGTTGATATGCATTTGCGTT

*********************************

OyamatsuTGCATCAAAAGCAACAACTTGACGAGGGAATGATTGGGACAAAGTAGAACTTCTTGTATA

AmpliconTGCATCAAAaGTAATAAGTTGACGAGGGAATGATTGAGATAAAGTAGAACTTCTTGTATA

*******************************************************

OyamatsuATGTATATCTTGAGAAGCAGCTGCTCCACCTGCTACAATTCCAAGAGCATAATGATATTC

AmpliconATGTATATCTTGAGAAGCAGCTGCTCCACCTGCTACAATTCCAAGAGCATAATGATATTC

************************************************************

OyamatsuAAGATCTAGTTTAATTTGATCTGGAGAATAAGTGTATGCCTTAATTCCATATAGCTTAAT

AmpliconAAGATCTAGTCTAATTTGATCTGGAGAATAAGTATATGCTTTGATTCCATACAGTTTAAT

******************************************************

OyamatsuGAAATGCTCATAATTACCTTGTCCAAACAAACTTAGGTAAAATAGTTTTAGGATTGAAGC

AmpliconGAAATGCTCATAATTACCTTGTCCAAACAAAGTTAGATAAAATAGTTTTAGGATTGAAGC

**********************************************************

OyamatsuAAGAGCTGCTGCACTTTTAGGAGGACAAGTAAAGTTACCACCAGCTCCCTACAAGGAAAG

AmpliconAAGAGCTGCTGCACTTTTAGGAGGACAAGTAAAGTTACCACCAGCTCC-TACAAGGAAAG

***********************************************************

OyamatsuCAATTGATTTTATACTTGAAGAACAATCTAGAAGATTGGAGAAATTCTCAAGAATTAGAA

AmpliconCAATTGATTTTATACTTGAAGAACATTCAAGAAGATTAGAGAAATTTTCAAGAATTAGAA

********************************************************

OyamatsuTTGCAACTTCTAGTCTCTCTAGTTTTTCTAGATTTAGATTTAGGTTGGCATCGAAATAAC

AmpliconTTGCAACTTCTAGTCTCTCTAGTTTTTCTAGATTTAGATTTAGGTTGGCATCGAAATAAC

************************************************************

OyamatsuTTGCATAATCTAGAAGTTCATCAACATCAAATGTACTTACATCAAATAGGAAAGGATTAG

AmpliconTTGCATAATCTAGAAGTTCATCAACATCAAATGTACTTACATCAAATAGGAA--------

****************************************************

OyamatsuGAGCAACCTGCA

Amplicon------------
